# Impaired K^+^ binding to glial glutamate transporter EAAT1 in migraine

**DOI:** 10.1038/s41598-017-14176-4

**Published:** 2017-10-24

**Authors:** Peter Kovermann, Margarita Hessel, Daniel Kortzak, Joanna C. Jen, Johannes Koch, Christoph Fahlke, Tobias Freilinger

**Affiliations:** 1 Institute of Complex Systems, Zelluläre Biophysik (ICS-4), Forschungszentrum Jülich, Jülich, Germany; 2 Departments of Neurology and Neurobiology, UCLA School of Medicine, Los Angeles, USA; 3 Department of Paediatrics, Salzburger Universitätsklinikum, Salzburg, Austria; 4 Department of Neurology and Epileptology, Hertie-Institute for Clinical Brain Research (HIH), Tübingen, Germany

## Abstract

*SLC1A3* encodes the glial glutamate transporter *h*EAAT1, which removes glutamate from the synaptic cleft via stoichiometrically coupled Na^+^-K^+^-H^+^-glutamate transport. In a young man with migraine with aura including hemiplegia, we identified a novel *SLC1A3* mutation that predicts the substitution of a conserved threonine by proline at position 387 (T387P) in *h*EAAT1. To evaluate the functional effects of the novel variant, we expressed the wildtype or mutant *h*EAAT1 in mammalian cells and performed whole-cell patch clamp, fast substrate application, and biochemical analyses. T387P diminishes *h*EAAT1 glutamate uptake rates and reduces the number of *h*EAAT1 in the surface membrane. Whereas *h*EAAT1 anion currents display normal ligand and voltage dependence in cells internally dialyzed with Na^+^-based solution, no anion currents were observed with internal K^+^. Fast substrate application demonstrated that T387P abolishes K^+^-bound retranslocation. Our finding expands the phenotypic spectrum of genetic variation in *SLC1A3* and highlights impaired K^+^ binding to *h*EAAT1 as a novel mechanism of glutamate transport dysfunction in human disease.

## Introduction

Glutamate is the major excitatory neurotransmitter in the central nervous system, and altered brain excitability caused by disturbed glutamate homeostasis plays a role in various paroxysmal neurological disorders^1–3^. Specifically, glutamate is a potent trigger of cortical spreading depression (CSD), the electrophysiological correlate of migraine aura^4^, and imbalance of glutamate release and clearance has been shown to underlie hemiplegic migraine (HM), a severe monogenic subtype of migraine with transient hemiparesis and other aura symptoms^5,6^.

EAAT1 is a glial glutamate transporter that contributes to glutamate clearance in the cerebral cortex, cerebellum, diencephalon and caudal brainstem^7^. Genetic variation in *SLC1A3* – the gene encoding EAAT1 - has been linked to several neurological disorders with partially overlapping clinical features^8–11^. In 2005, Jen *et al*. reported a *SLC1A3* missense mutation in a child with a complex syndrome comprising episodic ataxia, prolonged hemiplegia with migraine and seizures^8^. Here, we searched for and identified a novel heterozygous *SLC1A3* mutation in a young man with a similar but less severe clinical phenotype with recurrent episodes of migrainous headache accompanied by transient hemiparesis. To characterize the functional effects of the newly identified mutation on transporter function and compare them with results on other *SLC1A3* mutations, we used both electrophysiology and biochemistry.

## Results

### Case history and genetic analysis

**Figure 1:**
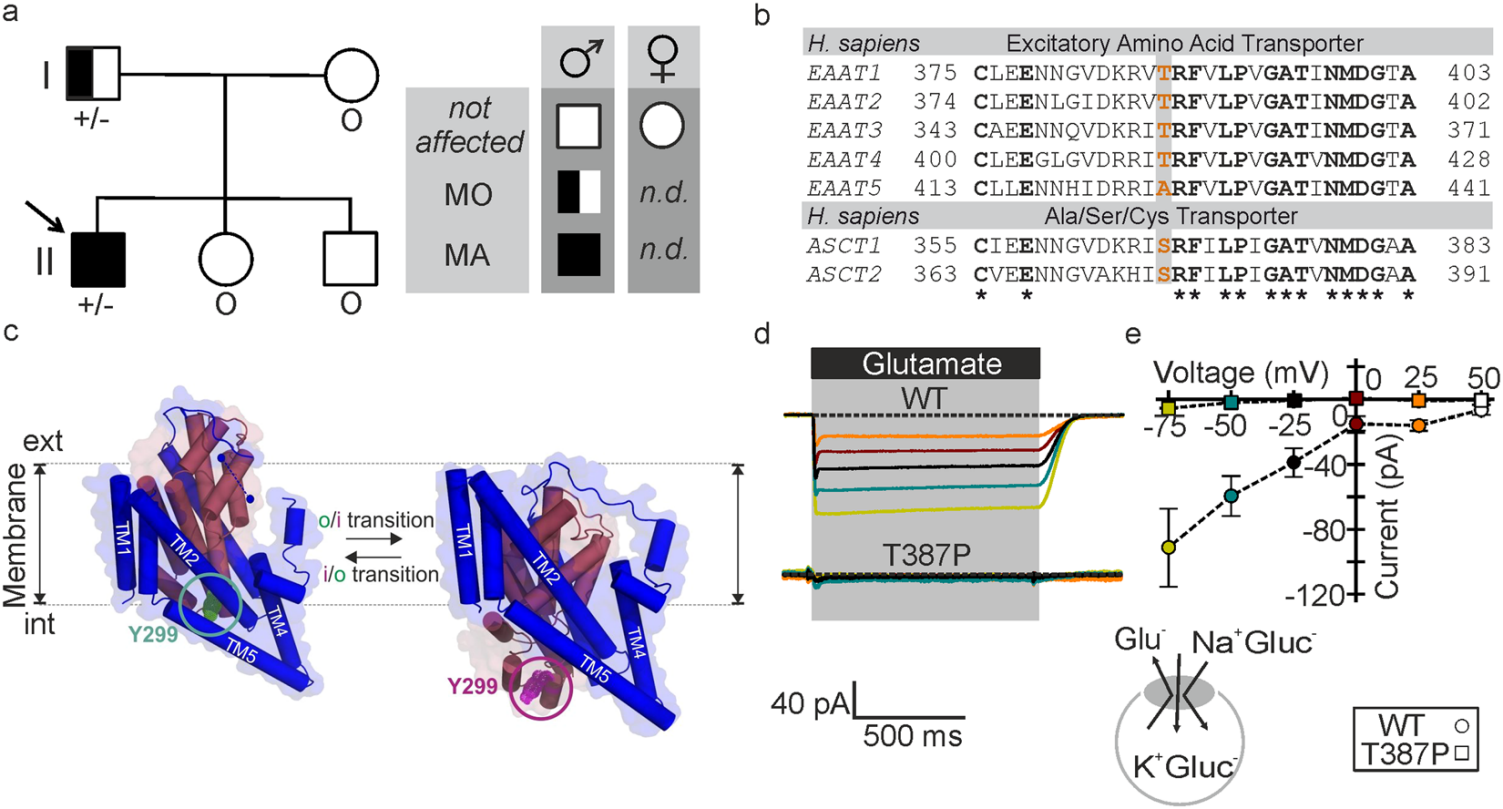
*SLC1A3* mutation causes the exchange of the conserved threonine 387 by proline in *h*EAAT1. (**a**) Pedigree of the kindred with *SLC1A3* ^*T387P*^ mutation. MA: migraine with aura (including hemiplegia); MO: migraine without aura. Arrow: index patient. Genotypes are indicated below each symbol (+/− denotes heterozygosity for *SLC1A3* ^*T387P*^ variant; o DNA not available). (**b**) On the protein level, the variant causes a threonine (ACC) to proline (CCC) change at position 387. Multiple alignment of human excitatory amino acid transporters (*h*EAATs) and neutral amino acid transporters (ASCTs) shows that the T387 homologue positions in the transporter isoforms are preferentially occupied by hydroxylated amino acids. (**c**) Position of the T387- homologue residue Y299 in the EAAT topology model in the outward (*o, green circle*, modified from 2NWX.pdb) and inward (*i*, *magenta circle*, modified from 4P3J.pdb) conformation (*dark purple*: trimerization domain; *dark red*: transport domain). (**d**) Representative current traces from whole-cell patch clamp recordings from HEK293T cells expressing WT (*top*) and T387P (*bottom*) *h*EAAT1 under uptake conditions with permeant anions substituted by gluconate. Pipette solution (in *mM*): 115 K-gluc, 2 Mg-gluc, 5 EGTA, pH 7.4; Bath solution: 140 K-gluc, 1 Mg-gluc, 2 Ca-gluc, 5 TEA, pH 7.4, ± 5 L-Glutamate. Glutamate perfusion is indicated by a horizontal black bar. **(e)** Current-voltage relationship from glutamate transport for WT (*circles*) and mutant (*squares, n* = 5/5) *h*EAAT1. Holding potentials used in the transport experiments are color-coded.

The patient is a now 22-year-old man from Serbia. Since age 11, he has suffered several episodes of severe migrainous headache with nausea and recurrent vomiting, accompanied by transient neurological deficits, including visual disturbances, prominent dysphasia and unilateral sensory and motor deficits; hemiparesis was reported to compromise his ability to hold/lift things. Headache was responsive to treatment with ibuprofen. Mild head trauma was reported as a triggering event in at least one attack. Retrospectively, the exact sequence and duration of neurological deficits could not be reliably determined. On three occasions, the patient presented to the hospital immediately after the onset of such attacks, and neurological evaluation at that time confirmed persistence of right-sided hemiparesis and dysphasia. Neuroimaging (both computed tomography and magnetic resonance imaging), performed during three attacks, was normal, while CSF (***C*** *erebro* ***s*** *pinal* ***f*** *luid*) analysis, performed during one attack, showed mild lymphocytic pleocytosis (9 cells/µl). EEG showed reversible left parieto-occipital slowing on one occasion, but was without evidence of seizure patterns. Interictal neurological examination was normal, and there was no evidence of episodic or permanent ataxia and no history of epileptic seizures. In subsequent years, the patient continued to experience similar attacks with severe headache with dizziness, mechanosensitivity, blurred vision, nausea and sometimes vomiting, but without associated neurological deficits. There was no evidence of headache attacks with focal neurological deficits in any other family member, but the patient’s father was found to suffer from migraine without aura (Fig. 1a).

The combination of migraine with unilateral motor deficits in at least some attacks phenotypically resembles hemiplegic migraine. In an initial screen of established HM genes (*CACNA1A*, *ATP1A2* and *SCN1A*)^12^ as well as in *PRRT2*, which was recently implicated in HM and other paroxysmal phenotypes^13^, mutations in any of these genes were ruled out. Sequencing of *SLC1A3* revealed a heterozygous nucleotide change c.1159 A > C (NM_004172). The variant was not detected in 100 control chromosomes nor listed in the databases dbSNP (*Short Nucleotide Variations Database*), ExAC (*Exome Aggregation Consortium*) and EVS (*Exome Variant Server*). Analysis of the parental DNA revealed the variant in the father, while DNA from other family members was not available (Fig. 1a). The variant c.1159 A > C predicts substitution of threonine 387 by proline (T387P). Thr387 is conserved in transmembrane helix 7 of mammalian EAAT1–4 transporters, but neither in EAAT5 nor in the related neutral amino acid exchangers (ASCTs) (Fig. 1b). EAAT glutamate transport is based on a large-scale rotational-translational movement of the substrate-harboring transport domain relative to the static trimerization domain^14,15^. Thr387 (Tyr299 in Glt_*Ph*_) is part of the transport domain and translates by 17 Å and rotates away from the trimerization domain during isomerization to the inward-facing conformation (Fig. 1c).

### T387P impairs *h*EAAT1-mediated glutamate transport

To study possible disease-associated changes in glutamate transport we expressed WT and T387P *h*EAAT1 in mammalian cells. Glutamate transport is associated with net charge transfer and can therefore be quantified by measuring glutamate-elicited currents^2^. In cells intracellularly dialyzed with K^+^-based solution in the absence of permeant anions, WT *h*EAAT1 generated robust currents upon L-glutamate application using a piezo-driven perfusion system, whereas no glutamate-elicited currents were observed in cells expressing T387P *h*EAAT1 (Fig. 1d,e).

### T387P abolishes *h*EAAT1-associated currents in the presence of internal K^+^

**Figure 2:**
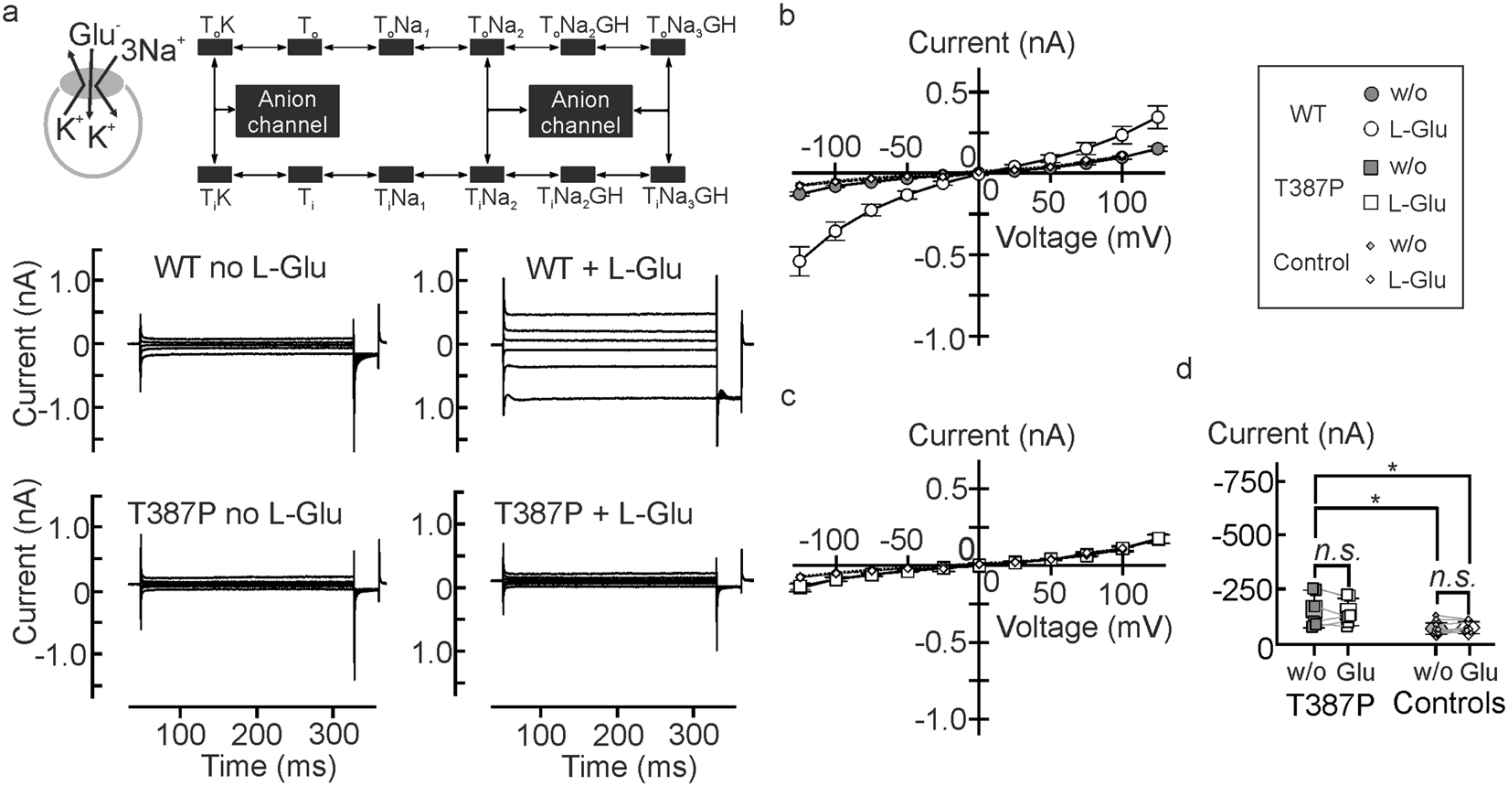
Internal potassium abolishes *h*EAAT1 anion currents in T387P *h*EAAT1. (**a**) Representative whole-cell patch clamp recordings from HEK293T cells expressing WT (*left*) or T387P (*right*) *h*EAAT1 internally dialyzed with K^+^-based solutions in the absence (*top*) and presence (*bottom*) of glutamate. Inset depicts the glutamate transport cycle^16,17,19^. (**b** and **c**) Current-voltage relationships for WT (**b**, *circles*), mutant (**c**, *squares*) *h*EAAT1, and untransfected HEK293T cells (**b** and **c**, *small diamonds*) under these experimental conditions (*n* = 9/12/8). (**d**) Statistical analysis of current amplitude differences from untransfected cells (*small diamonds*) and T387P *h*EAAT1 transfected HEK293T cells (*squares*) at a holding potential of −125 *mV* under uptake conditions. Bath solution (in *mM*): 140 NaNO_3_, 4 KCl, 2 CaCl_2_, 1 MgCl_2_, 5 HEPES, 5 TEA-Cl, pH 7.4; Pipette solution: 115 KNO_3_, 2 MgCl_2_, 5 EGTA, 5 L-glutamate, 10 HEPES, pH 7.4.

**Figure 3:**
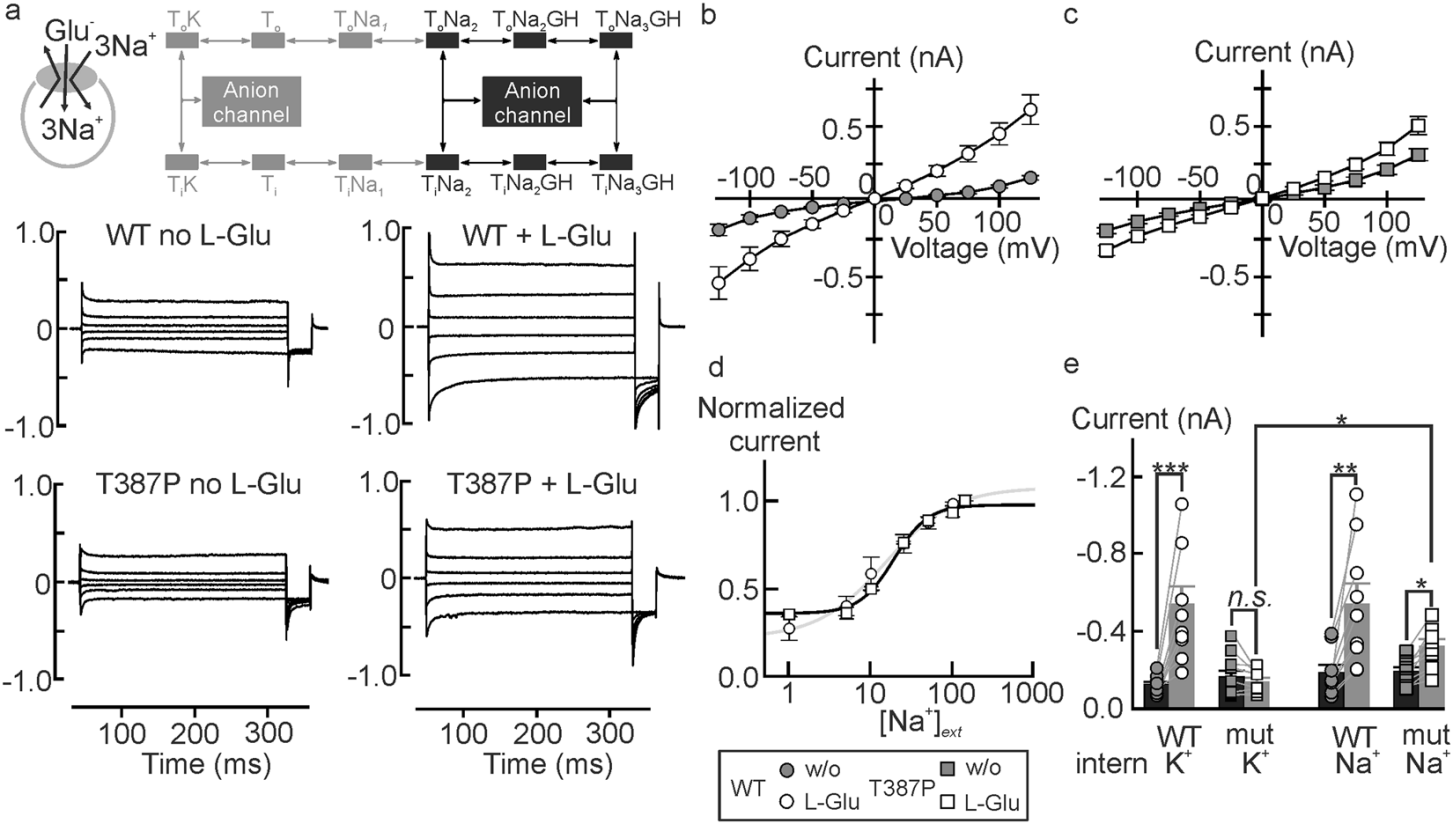
T387P *h*EAAT1 anion currents in the presence of internal Na^+^. (**a**) Representative current traces from whole-cell patch clamp recordings from HEK293T cells expressing WT (*left*) or T387P (*right*) *h*EAAT1 internally dialyzed with Na^+^-based solutions in the absence (*top*) and presence (*bottom*) of glutamate. Inset depicts the states within the glutamate transport cycle the transporter can assume during glutamate application in these experiments. (**b** and **c**) Current-voltage relationships for WT (**b**, *circles*) and mutant (**c**, *squares*) *h*EAAT1 under these experimental conditions (*n* = 9/9). Bath solution (in *mM*): 140 NaNO_3_, 4 KCl, 2 CaCl_2_, 1 MgCl_2_, 0.1 L-Glutamate, 5 HEPES, 5 TEA-Cl, pH 7.4; Pipette solution: 115 NaNO_3_, 2 MgCl_2_, 5 EGTA, 5 L-glutamate, 10 HEPES, pH 7.4. (**d**) Sodium dependences of WT (*circles*) and T387P (*squares*) *h*EAAT1 anion currents were determined by external sequential perfusion with solutions, in which NaNO_3_ was equimolarly substituted with CholineNO_3_ in the presence of 5 *mM* L-glutamate. (**e**) Mean current amplitudes at −125 *mV* for WT (*circles, black bars*) and mutant (*squares, grey bars, n* = 4/5) *h*EAAT1 anion currents under several experimental conditions as indicated.

EAATs assume anion-conducting conformations from certain intermediate states of the transport cycle^16,17^, and EAAT anion currents therefore represent a simple initial test which steps in the transport cycle are affected by T387P. The presence of the more permeant NO_3_^−^ as the main anion increases current amplitudes and permits characterization of EAAT anion currents with negligible contributions of uptake currents^18^. Figure 2a shows representative whole-cell current responses to voltages between −125 *mV* and +125 *mV* from HEK293T cells internally dialyzed with a K^+^- based solution expressing WT or T387P *h*EAAT1. Application of 0.1 *mM* L-glutamate resulted in a fourfold increase of the WT *h*EAAT1 current amplitude at -125 *mV* (*P*_*U*_ < 0.001, *d*_*Co*_ = 0.82) (Figs 2a,b and 3e). T387P causes a profound reduction of *h*EAAT1 anion currents under these conditions and abolished their substrate dependence (*P*_*U*_ = 0.59, *d*_*Co*_ = 0.20, *n* = 9/11) (Fig. 2a,c,d and 3e). T387P *h*EAAT1 currents were slightly larger than background (*P*_*t*_ = 0.024 (*w/o* L-glu)/0.013 (0.1 *mM* L-glu), Fig. 2c,d, see online supplementary text) indicating some residual activity under these conditions.

Glutamate is cotransported into the cell together with 3 Na^+^ and 1 H^+^, followed by the K^+^- bound re-translocation of the transporter back to the outward-facing state^2^. The two rate-limiting steps permit identification of two half-cycles, often denoted as Na^+^- and K^+^ hemicycles (Figs 2a and 3a). To separate T387P effects on these hemicycles we measured WT and mutant anion currents with Na^+^ as main internal cation^19^. Under these conditions, anion currents displayed a different time and voltage dependence than with internal K^+^, reflecting the tight coupling of anion channel gating to the transport cycle. With internal Na^+^, L-glutamate increased WT current amplitudes threefold (*P*_*U*_ = 0.005, *d*_*Co*_ = 0.64). T387P anion currents resemble WT currents in its time and voltage dependence, but are slightly smaller in amplitude. L-glutamate increased mean current amplitudes of the mutant significantly, but less efficiently than WT (*P*_*U*_ = 0.006, *d*_*Co*_ = 0.83) (Fig. 3a–c,e). Figure 3d shows the external sodium dependence of WT and mutant anion currents with internal Na^+^, demonstrating indistinguishable relative Na^+^ -dependences.

For WT as well as for T387P *h*EAAT1, we tested block of NO_3_^−^ currents by 100 *µM* DL-TBOA^20^ with both K^+^_*int*_ (*P*_*paired-t*_ = 0.002/0.049, *d*_*Co*_ = 0.92/0.22, *n* = 4/3, WT/T387P) or Na^+^_*int*_ (*P*_*paired-t*_ = 0.005/0.015, *d*_*Co*_ = 0.87/0.73, *n* = 5/4, WT/T387P) (Supplementary Fig. S1). In all cases, application of TBOA resulted in comparable background current amplitudes, indicating that our experimental procedure permits measurements of EAAT anion currents in isolation under all applied conditions.

### T387P impairs K^+^ association to the inward-facing transporter

**Figure 4:**
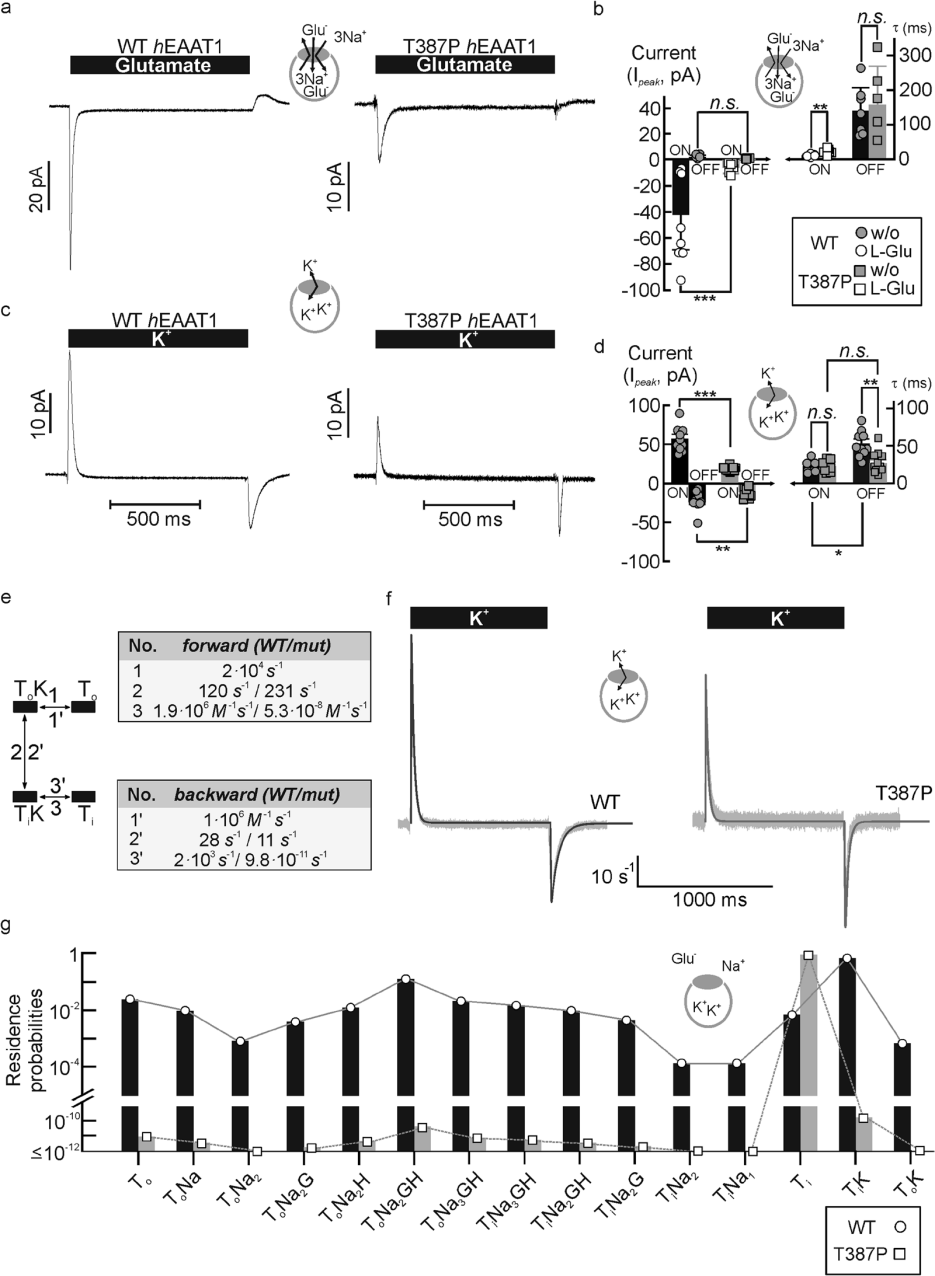
Impaired potassium association prevents glutamate uptake by T387P *h*EAAT1. (**a**) Representative current responses of HEK293T cells dialyzed with a solution containing (in *mM*) 115 Na^+^-gluconate and 5 L-glutamate and expressing WT (*left*) or T387P (*right*) *h*EAAT1 to rapid application of glutamate (Na^+^-glutamate exchange conditions). (**b**) Mean *ON* and *OFF* peak amplitudes and mean relaxation time constants (*τ*_*ON*_, _*OFF*_) from experiments shown in **a** (*n* = 9/7). (**c**) Representative current responses to rapid application of K^+^ to cells dialyzed with a K^+^-gluconate-based internal solution (K^+^-exchange conditions). In all of these experiments permeant anions were equimolarly substituted with gluconate in intra- (in *mM*, 115 K/Na-gluc, 2 Mg-gluc, 5 EGTA, pH. 7.4, 0/5 L-glutamate) and extracellular solutions (140 K/Na-gluc, 1 Mg-gluc, 2 Ca-gluc, 5 TEA, pH 7.4, ± 0/5 L-glutamate). (**d**) Mean *ON* and *OFF* peak amplitudes and relaxation time constants (*τ*_*ON*_, _*OFF*_) for the experiments illustrated in **c** (*n. a. ~ not analyzed; n. s. ~ not significant, n* = 8/11). (**e**) State diagram for the glutamate uptake cycle with highlighted four-state potassium hemicycle and list of fitting results for simulated currents from WT (*top*) and T387P (*bottom*) *h*EAAT1. (**f**) Simulated currents (solid lines) are shown with their underlying template recordings (*light grey*). (**g**) Simulated residence probabilities WT (*black bars*, *circles, solid lines*) and mutant EAAT1 (*grey bars*, *squares, dashed lines*) calculated from the data given in (**e**).

To further delineate transport steps that might be modified by T387P we used fast substrate application using a piezo-driven solution exchange under different ionic conditions (Fig. 4)^19,21–24^. We initially performed such experiments with cells dialyzed with a Na^+^-containing solution supplemented with saturating [L-glutamate]. These conditions – the so-called *exchange mode* - abolish forward glutamate transport, but permit glutamate transporter translocation upon rapid changes in external [L-glutamate]. These conformational changes give rise to capacitive currents^25,26^ (Fig. 4a,c,f) that permit quantification of the translocation process. The time course of current relaxation depends on the speed and the probability of translocation, whereas the amplitude further depends on the number of transporters in the membrane^26^. A comparison of *τ*_ON_ between transient currents of glutamate uptake and Na^+^-exchange conditions showed no differences (Glu: *P*_*t*_ = 0.06; Na: *P*_*t*_ = 0.093, *n* = 8/6; Figs 1d, 4a). WT and mutant currents differed in peak currents (*P*_*U*_ < 0.001, *d*_*co*_ = 0.58), and there are slight differences in their time dependence (*P*_*t*_ = 0.005, *d*_*Co*_ = 0.65, Fig. 4a,b). For fast application of L-glutamate we used a high concentration (5 *mM*) to permit rapid establishment of saturating [L-glutamate]. This maneuver significantly delays the complete substrate removal that is necessary because of the high substrate affinity of the transporters, preventing a meaningful analysis of current responses upon substrate-removal.

To test transitions within the K^+^ hemicycle we used rapid application of K^+^ to cells internally dialyzed with K^+^-gluconate-based solutions. Under these conditions application of 140 *mM* K^+^ results in a capacitive current due to transport domain translocation^26^. For WT *h*EAAT1, the complete charge movement is recovered upon stepping back to K^+^- free solution, albeit with a slower time constant (Fig. 4c,d). This behavior reflects the strict requirement for EAAT transporter re-translocation in the K^+^-bound conformation. Without external K^+^, there is only translocation possible from the inward to the outward-facing conformation, so that relaxation takes longer than for conditions with external K^+^. The different time courses of the *ON* and *OFF* capacitive currents result in differing peak current amplitudes (*P*_*U*_ = 0.001; *d*_*Co*_ = 0.42, Fig. 4c,d). For T387P *h*EAAT1, the time dependence of the transient *ON*-current (upon K^+^ application) resemble WT results (*P*_*t*_ = 0.94; Fig. 4d). However, mutant *OFF* time courses are faster than WT *OFF* time courses (*P*_*t*_ = 0.002) and similar to mutant *ON* time courses (*P*_*paired-t*_ = 0.42, Fig. 4d). This similarity is not caused by the limited time resolution of our solution exchange system, since amplitudes of *ON* and *OFF*- capacitive currents were similar for T387P, but different for WT transporters (Fig. 4c,d).

To quantify the T387P-induced changes in the K^+^ hemicycle we fitted capacitive currents under K^+^ transport conditions to the glutamate transport scheme using a genetic algorithm (Fig. 4e,f). Under these conditions transporters can only assume four different states, either in the apo conformation (*Ti*, *To*) or with bound potassium (*TiK*, *ToK*). For T387P *h*EAAT1 we obtained negligible K^+^ association rates to *Ti* and slightly altered translocation rates (Fig. 4e). We then inserted these rates into a published kinetic model to predict the steady-state probabilities that WT and mutant transporter resides in certain transport cycle states (Fig. 4g, and Supplementary Fig. S1). There are only small differences in steady-state residence probabilities under K^+^-transport conditions (Supplementary Fig. S2a), illustrating slow K^+^-association and -dissociation from *Ti*. This largely unaltered distribution explains the similarity in WT and mutant peak current amplitudes of K^+^ -induced capacitive currents under conditions (Fig. 4c,f). Figure 4g depicts residence probabilities under forward glutamate transport conditions. In the absence of external L-glutamate T387P *h*EAAT1 accumulates in *Ti* (Supplementary Fig. S2b). Slow Na^+^-bound inward translocation is still possible in mutant transporters, however, impaired K^+^-binding prevents re-translocation to *To*. Application of glutamate promotes inward translocation and results in the exclusive presence of mutant *h*EAAT1 in *Ti*. Thus, no L-glutamate association is possible for the mutant transporter, causing the absence of transport, anion currents and capacitive currents upon glutamate application in the mutant (Figs 1 and 2).

### T387P impairs the number of hEAAT1 in the surface membrane

**Figure 5:**
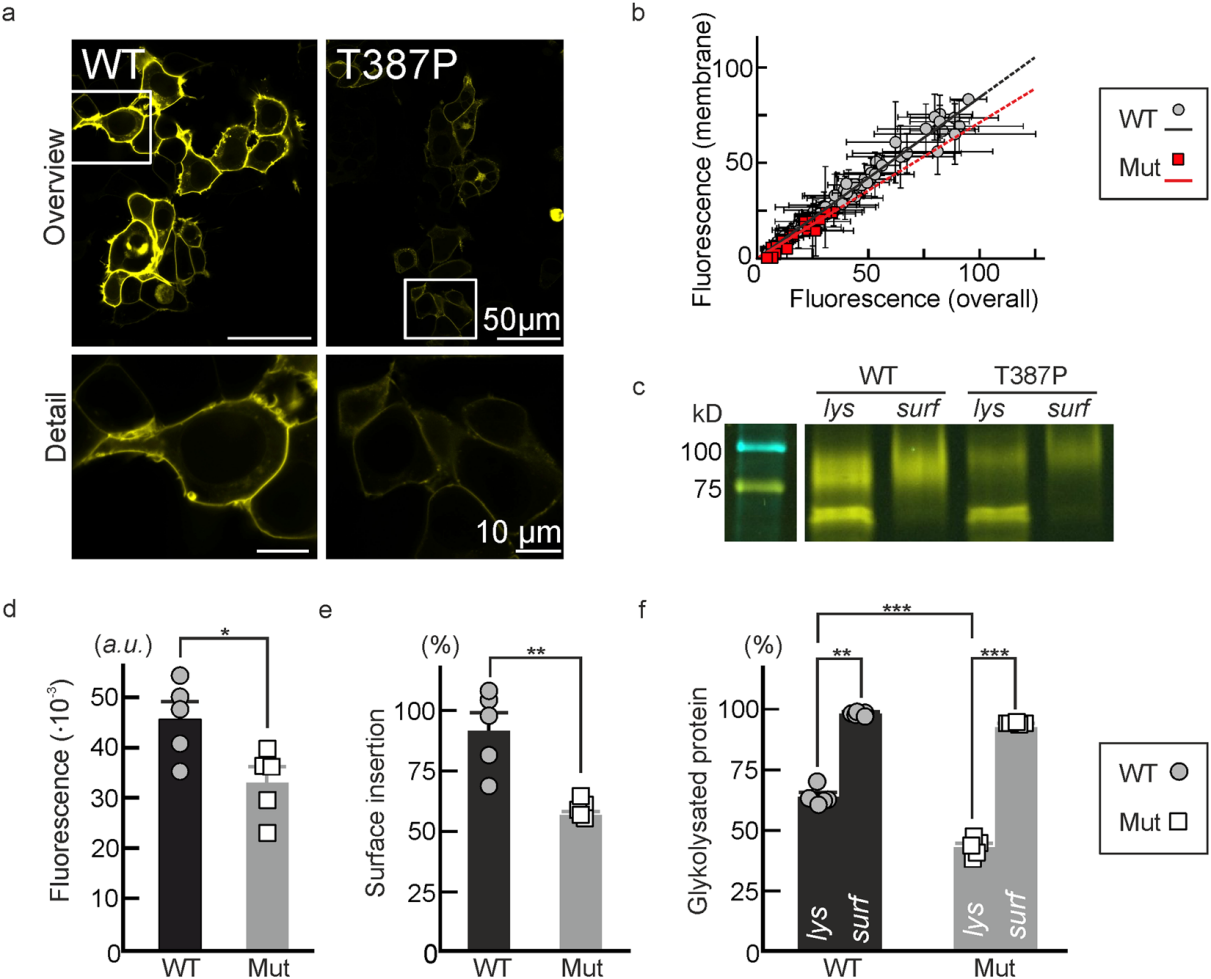
T387P decreases *h*EAAT1 transporter density in the surface membrane. (**a**) Representative confocal images from HEK293T cells expressing WT (*left*) or T387P (*right*) *h*EAAT1-YFP fusion proteins. (**b**) YFP-fluorescence distributions (*grey values*, *a. u*.) from WT (*grey circles*, *black line*, *n* = 72) and mutant (*red squares*, *red line*, *n* = 24) YFP-fusion proteins from HEK293T cells. Linear fits to the values point to slightly different surface insertion ratios for WT and mutant EAAT1 (slopes_*WT/T387P*_: 0.84/0.71; *R* ^2^_*WT/T387P*_: 0.97/0.91) (**c**) Representative cropped region of a SDS-PAGE showing YFP-fluorescence from total lysate (*lys*) or surface biotinylated protein (*surf*) for WT and T387P *h*EAAT1-YFP. MWL = Molecular Weight Ladder, BioRad-Precision plus, Dual color, #1610374. The corresponding full-length gel is provided as Supplementary Fig. 3. (**d**) Pooled total YFP- fluorescence emissions from WT (*circles*) and T387P *h*EAAT1-YFP (*squares, n* = 5/5). (**e**) Statistical analysis of surface biotinylation from experiments as shown in **c** indicates a lower ratio for surface expression of expressed protein for the mutant (*P*_*t*_ = 0.011, *n* = 5/5). (**f**) Surface insertion probability for core- and complex-glycosylated WT (*black bars*) and T387P (*grey bars*) protein.

We next quantified the effects of T387P on protein expression levels and subcellular distribution (Fig. 5). Confocal images show almost exclusive insertion of WT *h*EAAT1-YFP into surface membrane or in domains in close proximity. Mutant fusion proteins also preferentially insert into the surface membrane, however, with reduced expression levels (Fig. 5a). Figure 5b shows plots of surface membrane fluorescence intensities versus whole cell fluorescences for cells expressing either WT (grey circles) or mutant (red squares) proteins. The slopes of such linear regressed data sets provide the surface insertion probability and demonstrate slightly reduced values for mutant *h*EAAT1.

We next employed surface biotinylation to quantify *h*EAAT1 trafficking with an alternative technique. SDS-PAGE analysis of whole cell lysates and biotinylated fractions (Fig. 5c) provide fluorescent fusion protein amounts in whole cells as well as in surface membranes. T387P reduces total protein expression to ~72% of WT level (*P*_*t*_ = 0.024) (Fig. 5c,d). Figure 5e depicts mean ratios of surface membrane inserted protein by total protein. The surface membrane inserted mutant protein is decreased to ~50%. *h*EAAT1 predominantly exists in the core- or in complex-glycosylated state^27^ resulting in two major fluorescent bands in SDS PAGE (Fig. 5c). The percentage of complex-glycolysated protein in whole cell lysates was decreased for T387P *h*EAAT1 (*P*_*t*_ < 0.001, *d*_*Co*_ = 0.95). For WT as well as for mutant transporters complex-glycosylated protein inserted almost completely into the surface membrane (Fig. 5c,f).

Oligosaccharide side-chains are sequentially processed to the complex-glycosylated form in the Golgi apparatus, and analysis of *h*EAAT1 glycosylation thus indicate that T387P modifies early steps in *h*EAAT1 processing to its complex-glycosylated form. Since only complex-glycosylated transporters are inserted into the surface membrane (Fig. 5c,f), this processing defect not only explains lower protein expression levels (Fig. 5b,d), but also the discrete reduction in the number of mutant transporter in the plasma membrane (Fig. 5a,e).

## Discussion

We here report a novel heterozygous *SLC1A3* missense mutation in a patient with recurrent attacks of severe headache accompanied by transient focal neurological deficits including hemiparesis. Mutational screening of genes implicated in overlapping phenotypes, in particular HM, had been negative. The new variant was absent from 100 control chromosomes and public databases, the affected amino acid residue (T387) is highly conserved (Fig. 1), and functional analysis revealed a clear loss-of-function of mutant *h*EAAT1 (Figs 2–4).

In a patient carrying another *SLC1A3* missense mutation^8^ migrainous headache was associated with a complex spectrum of neurological symptoms. Our patient manifests with hemiplegic migraine without ataxia or seizures, and the phenotype of his father (also a mutation carrier) has even less severe manifestations with migraine without aura. Although there were references in the medical records of a history of “migraine” in other relatives, no other family members except for the patient’s parents could be reached for evaluation. Moreover, only the DNA from parents was available for genetic analysis, preventing a meaningful co-segregation analysis. The father, who was also carrier of the T387P mutation, suffered from migraine without aura, possibly reflecting reduced penetrance, as has been reported in paroxysmal phenotypes such as HM^28–30^.

Detailed analysis of the functional consequences of the T387P mutation revealed loss-of-function of *h*EAAT1 function with physiological internal K^+^ (Figs 1 and 2), whereas WT and mutant transporters resembled each other functionally in cells with Na^+^-based internal solutions (Fig. 2). Analysis of capacitive currents elicited by changes in external K^+^ with K^+^ as only cationic substrate identified impaired K^+^ association as molecular basis of T387P *h*EAAT1 transporter dysfunction. Fitting rate constants of a glutamate transporter kinetic scheme to these currents revealed dramatically impaired K^+^ binding to the inward-facing transporter and faster K^+^-bound translocation. Such alterations will result in an accumulation of transporters in the inward-facing conformation and abolish transport and channel function in the presence of internal K^+^ (Fig. 4). Since the Na^+^ hemicycle is less affected, WT and mutant currents are similar in the absence of internal K^+^ (Fig. 3). Biochemical analysis demonstrated that T387P additionally impairs trafficking, but leaves the surface insertion probability mainly unaffected. This alteration decreases total expression levels and the number of mutant transporters in the surface membrane (Fig. 5), resulting in a further reduction of mutant currents under all tested conditions.

Thr387 might directly contribute to K^+^ binding, or the mutation might impair formation of binding sites necessary for K^+^ association from the cytoplasm. At present, the molecular basis of K^+^ binding to EAAT/Glt_Ph_ is insufficiently understood. The current concept is that there is only one K^+^ binding site in the transport domain of the transporter^26,31^. K^+^ movement across the membrane is not based on conformational changes of the transport domain, but rather on a movement of the complete transport domain^2^. Our results are inconsistent with such a model. Whereas T387P has only minor effects on K^+^ binding to the outward-facing transporter, it causes a dramatic reduction of K^+^-association as well as -dissociation rate constants for the inward-facing *h*EAAT1 (Fig. 4). Our data would suggest the existence of multiple K^+^ binding sites within the transport domain and the consecutive occupation of these sites during translocation. Alternatively, T387P may modify closure of HP2 after K^+^ association and thus prevent K^+^-bound re-translocation. EAAT/Glt_Ph_ translocation is only possible when the two hairpin loops (HP1 and HP2) are closed^2^. T387P-mediated changes in HP2 dynamics could also account for some minor alterations of mutant transporters that were observed under exchange conditions (Fig. 4,b).

Our clinical and functional data re-inforce the concept that disturbed glutamate homeostasis from genetic variations in *SLC1A3* could lead to a broad spectrum of neurological manifestations with overlapping features. The first *SLC1A3* mutation (P290R)^8^, arose *de novo* in a single patient with episodic ataxia, hemiplegia with migraine, and epilepsy, impairs glutamate transport and enhances *h*EAAT1 anion currents^27,32^, likely reducing glial intracellular [Cl^−^]^33,34^. Another *SLC1A3* mutation, found in multiple members of a family with episodic ataxia (C186S), was reported to slightly reduce glutamate uptake levels^9^ and to modify intracellular transport of EAAT1^35^, while *h*EAAT1 anion currents were not studied. A sequence variant predicting E219D, which was found in some individuals with Tourette syndrome^10^, increases the relative surface membrane insertion probability of *h*EAAT1, predicting gain-of-function of glutamate transport and anion channel activity. Gene duplication in *SLC1A3* in patients with ADHD and/or autism-like features is also expected to increase *h*EAAT1 glutamate transport and anion currents^11^. In contrast to these published mutations, the *SLC1A3* ^T387P^ mutation results in loss-of-function of glutamate transport and the loss of anion channel activity.

Glutamate is an important trigger of CSD, the correlate of migraine aura. Assuming that the episodes of transient hemiparesis and other neurological deficits in our patient are functionally related to aura events, impaired glutamate reuptake by functionally altered *h*EAAT1 is likely to increase susceptibility to these episodes. Accurate prediction of the extent of glutamate accumulation at the synapse caused by the mutation is currently not possible. Glial cells of the heterozygous patient are expected to express both WT and mutant transporter subunits, and the majority of trimeric *h*EAAT1^36,37^ will contain WT as well as T387P *h*EAAT1 subunits. Individual subunits function independently of each other, and the localization of Pro387 does not predict altered interaction of mutant with WT subunits. Reduction of glutamate uptake in affected individuals will thus critically depend on the ratio of WT and mutant subunits in native cells, a parameter that we cannot determine at the moment. If WT and mutant subunits were expressed at identical levels, a reduction of *h*EAAT1-mediated glutamate uptake to about 50% would be expected. However, since T387P reduces the number of translated mutant *h*EAAT1 subunits (Fig. 5), this reduction might be even less pronounced. Taken together, our findings suggest that the mutation will only have a discrete effect on glutamate homeostasis in the affected patient. This illustrates how delicately glutamate concentrations have to be controlled in the human brain. Our result might explain the rather benign clinical course of the index patient as well as the phenomenon of reduced penetrance in his father.

In summary, our observation expands the phenotypic spectrum associated with genetic variation in *SLC1A3*, and our functional data on mutant *h*EAAT1 highlight distinct glutamate transporter dysfunctions in each of the reported paroxysmal neurological syndromes.

## Methods

### Patients and genetic analysis

In the index patient, direct sequencing was used to identify mutations in the already established genes associated to glutamate imbalance related paroxysmal disorders like episodic ataxia and hemiplegic migraine (*CACNA1A*, *ATP1A2* and *SCN1A*) as well as *PRRT2*, which was recently implicated in HM and other paroxysmal phenotypes^13^. Subsequently, all exons and exon-intron boundaries of *SLC1A3* were subjected to direct sequencing as previously described^8^. Targeted sequencing of the novel *SLC1A3* variant was also performed in DNA samples from the parents and from 50 healthy control individuals. Novelty of the newly identified mutation was verified by database queries in dbSNP (Short Nucleotide Variations database; https://www.ncbi.nlm.nih.gov/snp), ExAC (***E*** *xome* ***A*** *ggregation* ***C*** *onsortium*) and EVS (***E*** *xome* ***V*** *ariant* ***S*** *erver*). Written informed consent for genetic analysis was obtained from all participants in line with an approval from the ethics committee of the medical faculty of the Ludwig-Maximilians-Universität, München (former affiliation of TF), and the index patient agreed with the publication of clinical and genetic details. All experiments were performed in accordance with the Declaration of Helsinki.

### Functional characterization of WT and mutant EAATs

The T387P mutation and YFP-fusion of *h*EAAT1 proteins were generated and subcloned into the vector pcDNA3.1 (Invitrogen) using PCR-based strategies^27,38^. HEK293T cells were transfected using the Ca_3_(PO_4_)_2_ technique or as described previously^18,27,38^ and whole-cell patch clamped using EPC10 (HEKA Electronic, Germany) or Axopatch 200B (Molecular Devices, USA) amplifiers and standard solutions as described^27,38^. Current-voltage relationships were constructed from steady-state current amplitudes (*I*_*ss*_) 250 or 750 *ms* after the voltage jumps or concentration exposures (Figs 1,2 and 3). Possible contaminations with non-EAAT anion channels were tested by blocking WT and mutant *h*EAAT1 anion currents with the non-transportable blocker DL-TBOA (DL-threo-β-Benzyloxyaspartate, 100 *µM*, Tocris, Bio-Techne, Germany)^20^ (see supplementary Fig. S1). For fast application of substrates, a piezo-driven system with a dual-channel theta glass tubing was used (Fig. 4) (MXPZT-300, Siskiyou, USA) (see online supplementary text). Current amplitudes (I_*peak ON, OFF*_) were calculated from maximal peak currents of capacitive currents. Mean relaxation time constants (τ_*ON, OFF*_) of capacitive currents were calculated from pooled time constants of mono-exponential fits to the relaxation transients.

The K^+^-dependence of *h*EAAT1 currents was simulated by solving differential equations to a four-state scheme (Fig. 4e, see supplementary text) using published values as starting values^19^. Rate constants were estimated by optimizing the model against the time courses of currents using the genetic algorithm as implemented in the Python package DEAP^39^.

### Confocal microscopy and biochemical characterization of EAAT fusion proteins

Confocal imaging was carried out on living cells as described (see supplementary text)^40^. Protein amounts were estimated by scanning SDS-PAGEs (10%) with a Typhoon^TM^ FLA9500 gel scanner (GE Healthcare, Sweden) and quantifying YFP- fluorescence with the Fiji gel analysis package. Surface expression of *h*EAAT1 was quantified with cell surface biotinylation as described previously (see supplementary text)^27^.

### Data analysis

Data were analyzed with a combination of Clampfit (Molecular Devices, USA), Patchmaster (HEKA, Germany), SigmaPlot (Jandel Scientific, USA), MATLAB (Mathworks, USA), Fiji (Open Source), and Excel (Microsoft Corp., USA) programs. All data are given as means ± SEM, otherwise stated in the text. For statistic evaluation two-tailed Student’s-*t*-tests (*t*), paired-*t*-tests (*paired-t*) or Mann-Whitney-*U*-tests (*U*) were used (*P* < 0.05) and indicated as subscripted indices added to the *P*-values. Effect sizes were calculated from *Cohen’s* coefficients (*d*_*Co*_)^41^ and assessed as *d*_*Co*_ < 0.2 (*no*), 0.2 ≤ *d*_*Co*_ < 0.5 (*weak*), 0.5 ≤ *d*_*Co*_ < 0.8 (*medial*) and 0.8 ≤ *d*_*Co*_ < 1.3 (*large*)^42^.

### Data Availability Statement

The datasets generated during and/or analyzed during the current study are available from the corresponding author on reasonable request.

## Electronic supplementary material

Supplementary Information
