# Supplementary material for: Impaired K+ binding to glial glutamate transporter EAAT1 in migraine
Source: Sci Rep. 2017 Oct 24;7:13913. doi: 10.1038/s41598-017-14176-4 (PMC5654970; doi:10.1038/s41598-017-14176-4)
Supplement: Supplementary file 1 — Supplementary Information [file 41598_2017_14176_MOESM1_ESM.pdf]

Impaired K^+ binding to glial glutamate transporter EAAT1 in migraine

Peter Kovermann[1], Margarita Hessel[1], Daniel Kortzak[1], Joanna C. Jen[2], Johannes Koch[3], Christoph Fahlke[1,§], and Tobias Freilinger[4,§,*]

[1]Institute of Complex Systems, Zelluläre Biophysik (ICS-4), Forschungszentrum Jülich, Jülich Germany, [2]Departments of Neurology and Neurobiology, UCLA School of Medicine, Los Angeles, USA, [3]Department of Paediatrics, Salzburger Universitätsklinikum, Salzburg, Austria, and [4]Department of Neurology and Epileptology, Hertie-Institute for Clinical Brain Research (HIH), Tübingen, Germany

[§] these two authors contributed equally to this work

Supplementary Text

Supplementary methods

Kinetic Modeling

The K^+- dependence of WT and T387P *h*EAAT1 currents was simulated by solving differential equations to the four-state kinetic scheme given in figure 4e. In these simulations we assumed that other states of the transport cycle were not affected by the T387P mutation and used published reaction rates from Bergles et al [1]. In all cases, the ratio of the products of the forward and backward rates of all transitions were kept constant to preserve detailed balance within the full kinetic scheme describing EAAT function[2]. Since our experiments revealed that T387P *h*EAAT1 is transport-incompetent (Fig. 1d, e), we restrained the amplitude of forward glutamate transport to negligible values.

Solution exchange

At the end of each fast solution exchange experiment we removed the cell from the patch pipette and measured dead times ($2\ ms <,\ \tau_{dead} < 26\ ms$, $\tau_{dead} = 10.4 \pm 5\ ms\ (\pm\ \sigma)$) and 20-80 rise times ($0.85 \leq \tau_{rise20\text{-}80} \leq 6.3\ ms$, , $\tau_{rise\ 20\text{-}80} = 2.6 \pm 1.5\ ms\ (\pm\ \sigma)$) of our system by fast application of the used solution to the open pipette[3].

Our analysis might be affected by the limited solution exchange speed of fast substrate application in the whole-cell configuration[2]. To assess the effect of this experimental limitation we assumed that $[K^+]_o$ changes exponentially after fast K^+ application or removal and tested the consequences of various K^+ exchange time constants on our fitting results. This analysis indicated a minimum of the sum of squares of regression residuals (*SSR*) at $\tau = 2\ ms$ for both WT and T387P in good agreement with the experimentally determined mean values of $\tau_{rise20\text{-}80} = 2\ ms$. The comparison of *SSR* revealed that the fitting results for K^+ -association/dissociation were rather independent from the actual potassium exchange rates and can be accurately

determined within values that we experimentally determined (see online supplementary figure 2).

Confocal microscopy and biochemical characterization of EAAT fusion proteins

To compare YFP- emission from WT and mutant fusion proteins we used identical detector sensitivities (Fig. 5a). Fluorescence levels of cell membranes and cytosolic fluorescences were analyzed separately with the open source software Fiji (http://imagej.nih.gov/ij/). We manually placed transects throughout individual transfected cells, and determined fluorescence intensities along these lines. WT and mutant transporters preferentially insert into the cell membranes thus generating fluorescence maxima at the cell surfaces, followed by lower cytoplasmic values. We averaged maximum fluorescence values from multiple cells and calculated mean surface membrane fluorescence intensities (F_{mem}) and standard deviations (σ). Mean cytosolic fluorescences (F_{cyto}) were calculated from regions of interest that were manually placed into the cytosolic regions of the cells. Resulting overall whole cell fluorescences from single cells ($F_{mem} + F_{cyto}$) expressing YFP-tagged transporters were plotted ($\pm \sigma$) against their membrane fluorescences F_{mem} ($\pm \sigma$), and fitted with linear functions (Fig. 5b).

To quantify surface expression HEK293T cells were transiently transfected with WT and T387P *h*EAAT1, and surface insertion was quantified by comparing the total YFP-fluorescence in whole cell lysates (*tp*) and surface biotinylated fractions (sf_{biotin}) for WT and for the mutant as $sf_{biotin} \cdot tp^{-1}$. Protein expression was measured by scanning SDS-PAGEs (10 %) with a Typhoon™ FLA9500 gel scanner (GE Healthcare, Uppsala, Sweden) and quantifying YFP-fluorescence with the open source gel analysis package provided by Fiji. A representative full-length gel is provided as supplementary figure 4.

Glycosylation ratios (*ratio*$_{glyc.}$) were estimated for each genotype individually from the ratios of YFP- fluorescences of the lower (*non/core-glycosylated*) and the upper (*complex glycosylated*) bands. The values were separately determined for whole cell lysates and surface biotinylated proteins (Fig. 5c, f).

Background currents and correction for endogenous K^+ currents. Untransfected cells exhibited only very small currents with (in *mM*) 140 $NaNO_3$ (*ext*) and 115 KNO_3 (*int*) that were glutamate independent (w/o L-glut, -66 ± 8.1 (*C. I.:* 17.9) *pA*, with L-glut, -70.1 ± 6.7 (*C. I.*: 15.5) *pA*, $P_U = 0.403$, $d_{Co} = 0.11$ (*n*), *n* = 11/9) (Fig. 2b-d).

HEK293T cells express endogenous K^+ channels that are not fully blocked by the added TEA. These channels generate a K^+ leak current that result in a rectangular current component upon fast changes of external [K^+]. We corrected for this endogenous current by adding or subtracting a rectangular spline to the measured current amplitudes.

1 Bergles, D. E., Tzingounis, A. V. & Jahr, C. E. Comparison of coupled and uncoupled currents during glutamate uptake by GLT-1 transporters. *J. Neurosci.* **22**, 10153-10162 (2002).

2 Colquhoun, D., Jonas, P. & Sakmann, B. Action of brief pulses of glutamate on AMPA/kainate receptors in patches from different neurones of rat hippocampal slices. *J. Physiol.* **458**, 261-287 (1992).

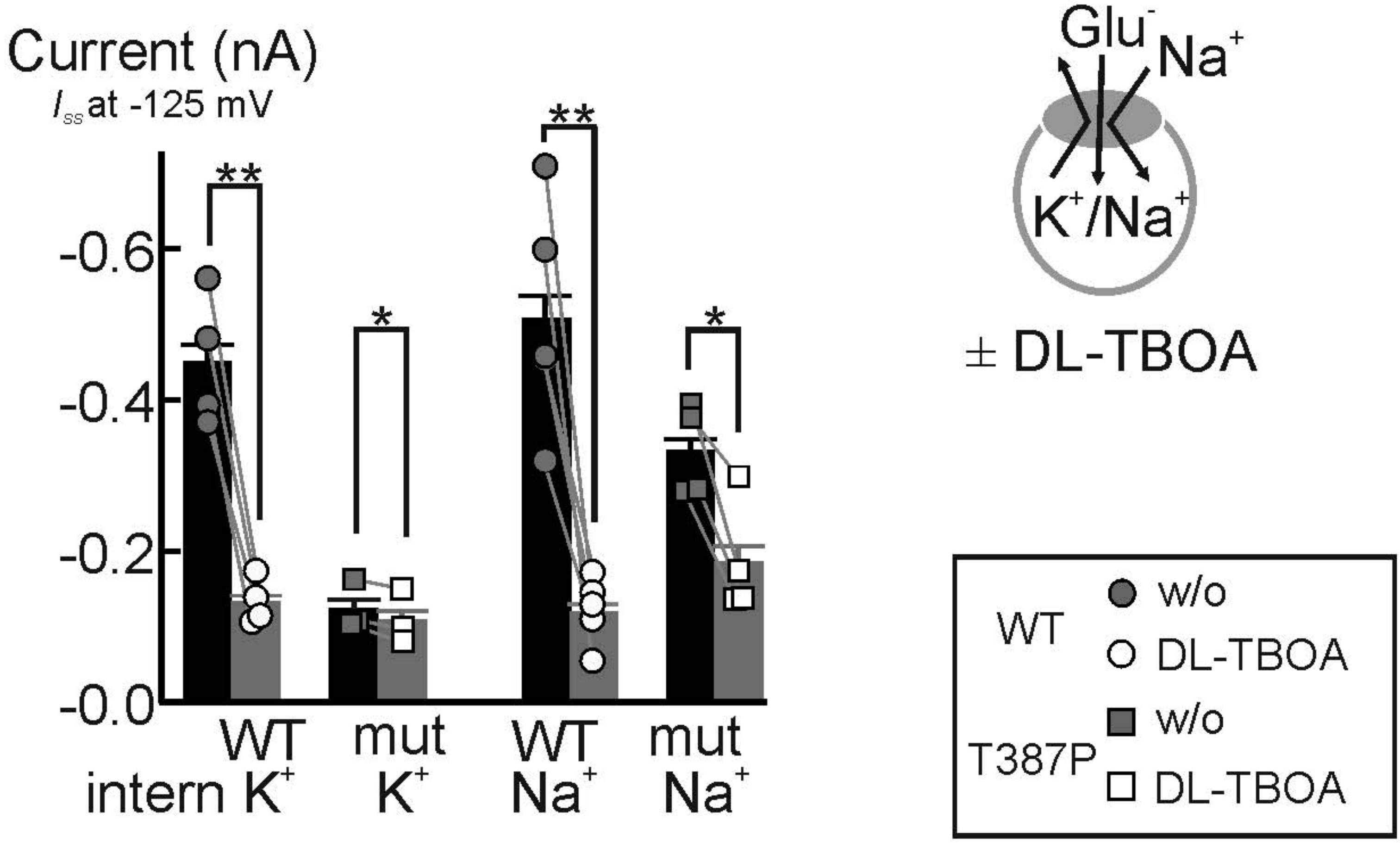

Supplementary Fig. S1

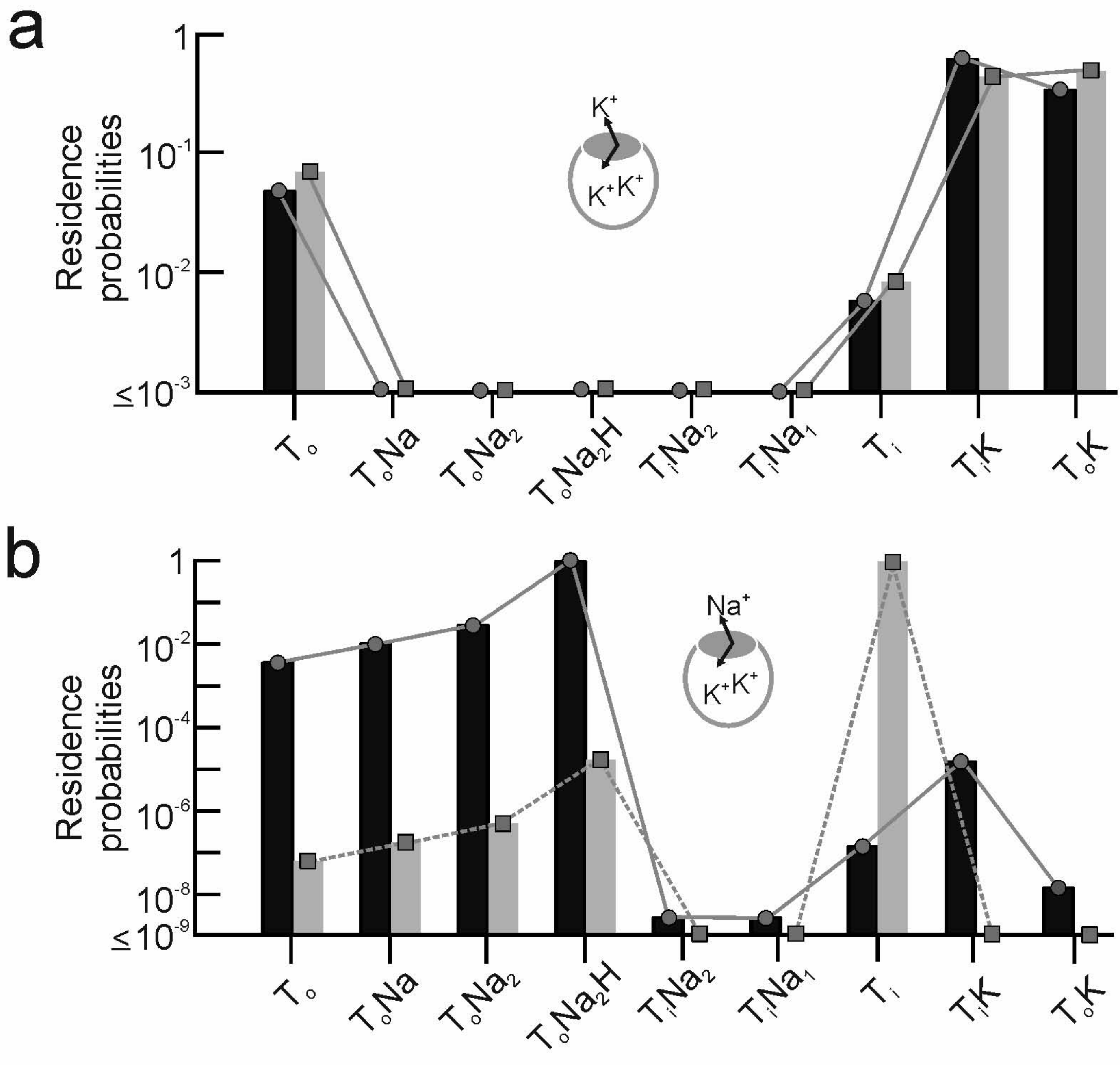

Supplementary Fig. S2

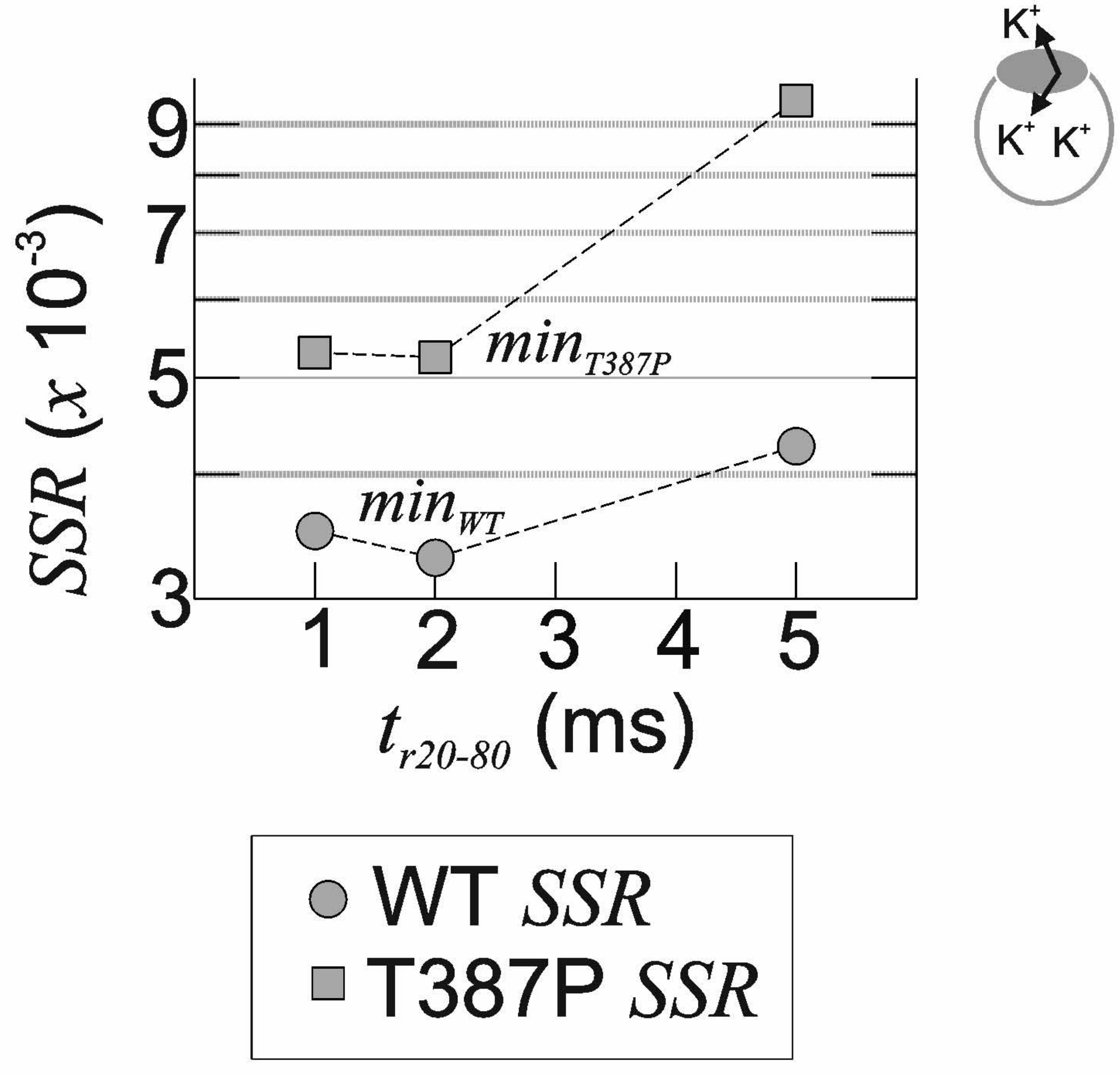

Supplementary Fig. S3

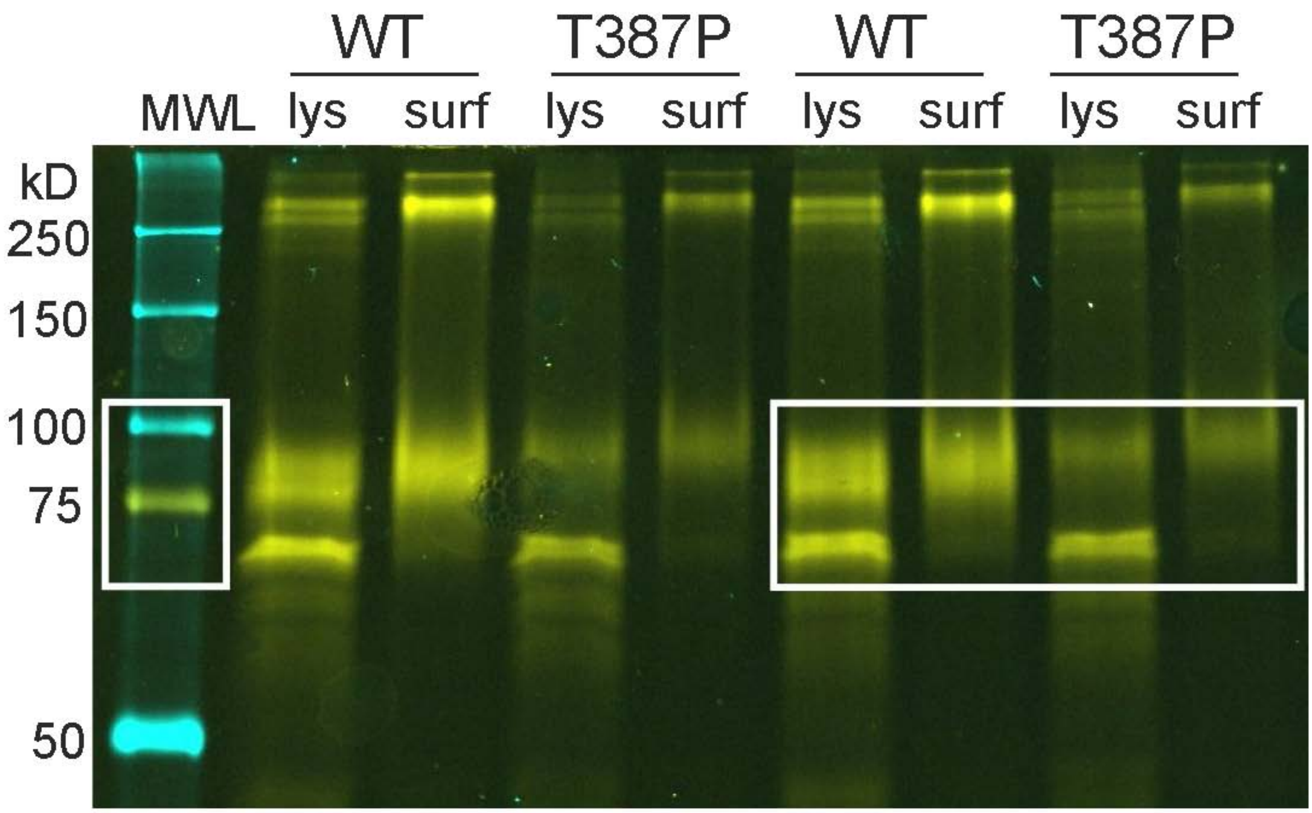

Supplementary Fig. S4
